# Prolonged SARS‐Cov‐2 shedding with rapid IgG antibody decay in a COVID‐19 patient: A case report

**DOI:** 10.1002/jcla.24002

**Published:** 2021-09-15

**Authors:** Jinbao Huang, Changqing Lan, Xinhang Wang, Mingxiang Huang

**Affiliations:** ^1^ Department of Respiratory Medicine The People's Hospital Affiliated to Fujian University of Traditional Chinese Medicine Fuzhou China; ^2^ Department of Radiology Fuzhou Pulmonary Hospital of Fujian Educational Hospital of Fujian Medical University Fuzhou China; ^3^ Department of Respiratory and Critical Care Medicine Fuzhou Pulmonary Hospital of Fujian Educational Hospital of Fujian Medical University Fuzhou China; ^4^ Department of Clinical Laboratory Fuzhou Pulmonary Hospital of Fujian Educational Hospital of Fujian Medical University Fuzhou China

**Keywords:** antibodies, coronavirus disease 2019, false negative of RT‐PCR, prolonged viral RNA shedding, SARS‐Cov‐2

## Abstract

**Background:**

The coronavirus disease 2019 (COVID‐19) epidemic is still spreading rapidly around the world. Recent cases with prolonged severe acute respiratory syndrome coronavirus 2 (SARS‐CoV‐2) RNA detection have been successively reported, and the phenomenon of false‐negative real‐time polymerase chain reaction (RT‐PCR) results of SARS‐CoV‐2 RNA or “repositive” was also described in COVID‐19 patients.

**Methods:**

We report a 69‐year‐old female patient with hypertension, suspected lung tumor, and previous history of total hysterectomy for hysteromyoma who presented with moderate COVID‐19 symptoms and was positive for SARS‐CoV‐2 RNA by RT‐PCR when she traveled from the USA to China.

**Results:**

The patient required second and third re‐hospitalizations due to “repositive” SARS‐CoV‐2 throat swab test results during post‐charge solitary isolation and observation, and serum SARS‐CoV‐2‐IgG decayed rapidly before disappearing on illness Day 139 when the throat swab was still positive. The virus shedding lasted for at least 146 days (the last positive throat swab test result was on illness Day 146, and the first true‐negative test result was on illness Day 151) since her initial positive test.

**Conclusion:**

Prolonged SARS‐CoV‐2 RNA viral shedding is prone to occur in an immunocompromised host, wherein changes in the host immune status can lead to repeated positive SARS‐CoV‐2 detection. Moreover, the SARS‐CoV‐2‐IgG may decrease rapidly and disappear before virus removal, indicating there may be certain limitations on the protective effect of the SARS‐CoV‐2 antibody, which deserves clinical attention.

## 1 INTRODUCTION

The coronavirus disease 2019 (COVID‐19) epidemic remains globally and rapidly widespread. As of March 8, 2021, severe acute respiratory syndrome corona virus 2 (SARS‐CoV‐2) infections have caused over 116 million cases and over 2.5 million deaths worldwide, continuing to worsen in most countries (https://www.who.int/publications/m/item/weekly‐operational‐update‐on‐covid‐19‐‐‐8‐march‐2021). As established, a positive SARS‐CoV‐2 RNA test is the gold standard for COVID‐19 diagnosis,^1^ and two consecutive negative SARS‐CoV‐2 RNA test results are one of the criteria for hospital discharge.^1^ A retrospective study of 301 COVID‐19 patients in China showed that the viral shedding median duration was 20 days from illness onset, whereas prolonged virus replication (>28 days) was found in 25 patients, with the longest duration at 42 days.^2^ Currently, some similar cases with prolonged SARS‐CoV‐2 RNA detection have been successively reported, with the longest shedding duration lasting from 55 to 156 days.^3^, ^4^, ^5^, ^6^, ^7^, ^8^, ^9^, ^10^, ^11^ Additionally, recent reports have also showed false‐negative real‐time polymerase chain reaction (RT‐PCR) results for SARS‐CoV‐2, which is known as the “repositive” (RP) phenomenon.^2^, ^12^, ^13^, ^14^ Herein, we report a case of a recovered COVID‐19 patient with repeating recurrence of positive SARS‐CoV‐2 RNA for at least 146 days (the last positive test result was on illness Day 146, and the first true‐negative test result was on illness Day 151), with findings of gradual anti‐SARS‐CoV‐2 IgG disappearance before virus removal. This study was approved by the Fuzhou Pulmonary Hospital of Fujian Ethics Committee, and the patient provided an informed consent for case publication.

## 2 CASE INTRODUCTION

**FIGURE 1:**
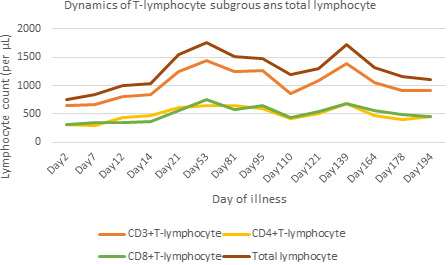
Dynamics of T‐lymphocyte subgroups and total lymphocyte counts after illness onset. After the first hospitalization treatment, T‐lymphocyte subgroups and total lymphocyte counts initially increased to normal, whereas the indexes repeatedly declined during the later course of the disease

**FIGURE 2:**
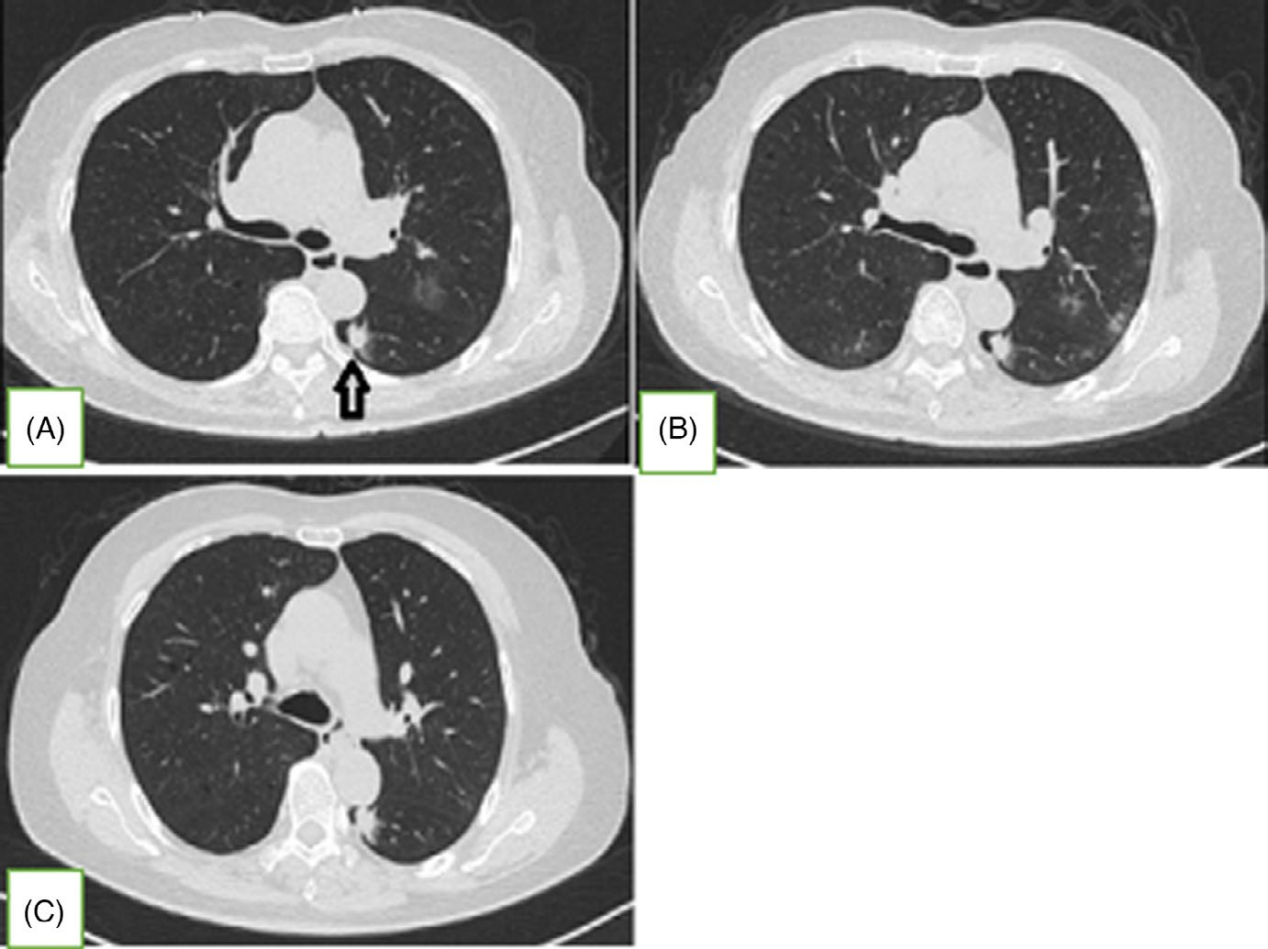
Dynamics of Chest CT findings after illness onset. (A) On illness day 2, chest CT findings showed multiple patchy ground‐glass opacities in both lungs, indicating viral pneumonia, and a nodular consolidation shadow (1.0 × 0.7 cm) with lobulated sign and spiculation in the dorsal lobe of the left lower lung (LLL‐S^6^), indicating suspected isolated lung tumor (arrow). (B) On illness day 10, repeated chest CT showed progress of partial lesions with absorption in both lungs, and the suspected cancerous nodule in the LLL‐S^6^ was similar to previous findings. (C) On illness day 32, repeated chest CT showed significant absorption of infected lesions in both lungs, and the initial solid nodule in the LLL‐S^6^ remained similar to previous findings

A 69‐year‐old Chinese female patient, who traveled from New York, USA, to Fuzhou Changle International Airport, China, on March 22, 2020, was positive for her throat swab SARS‐CoV‐2 RNA RT‐PCR test on the same day during routine screening. She reported to have felt slight fatigue and loss of appetite, without cough, fever, dyspnea, or other infectious symptoms. After probing for her history of hypertension and total hysterectomy for hysteromyoma, she was subsequently admitted to the negative pressure isolation room in the Fuzhou Pulmonary Hospital of Fujian Province, a designated COVID‐19 hospital, on the next day. Main laboratory examination findings throughout the disease course after onset are shown in Tables 1, 2, 3 and Figure 1. Her throat was swabbed and tested again using qualitative RT‐PCR assay, which was performed using a COVID‐19 nucleic acid detection kit (Da An Gene Co., Ltd. of Sun Yat‐sen University), reporting a definitely positive SARS‐CoV‐2 RNA test. Increased results of cardiac‐associated enzymes [creatine kinase (CK), creatine kinase isoenzyme (CK‐MB), and myohemoglobin (MYO)] and interleukin‐6 (IL‐6) were shown on admission. On peripheral blood analysis, leukopenia, thrombocytopenia, and lymphopenia with obviously decreased T‐lymphocyte subgroups, including CD3^+^, CD4^+^, and CD8^+^ T cells, were also noted. Arterial blood gas analysis showed an oxygen partial pressure of 80.6 mm Hg, a carbon dioxide partial pressure of 36.9 mm Hg, and an arterial oxygen saturation of 94.6%. Additionally, C‐reactive protein (CRP), erythrocyte sedimentation rate (ESR), and lactic dehydrogenase (LDH) levels gradually increased during hospitalization. Her human immunodeficiency virus antibody test was negative and procalcitonin (PCT) kept normal. Moreover, chest CT findings showed multiple patchy ground‐glass opacities in both lungs, indicating viral pneumonia, and a nodular consolidation shadow (1.0 × 0.7 cm) with lobulated sign and spiculation in the dorsal lobe of the left lower lung (LLL‐S^6^), indicating suspected isolated lung tumor (Figure 2A). An additional bedside echocardiography showed aortic sclerosis, aortic valve thickening, normal left ventricular systolic function, and tricuspid regurgitation with mildly elevated pulmonary arterial pressure. Color Doppler ultrasonography of the whole abdomen revealed double renal cyst without other abnormalities; however, color Doppler ultrasonography of the neck suggested the possibility of nodular goiter without lymph node enlargement.

**TABLE 1:** Dynamics of laboratory indexes

Day of illness	IL−6, pg/ml	CRP, mg/L	ESR, mm/h	WBC, ×10^9^/L	NEU, ×10^9^/L	PLT, ×10^9^/L	LYM, ×10^9^/L	SaO_2_, %	LDH, U/L	CK, U/L	CK‐MB, ng/ml	TYO, ng/ml
2 (March 23rd, 2020):	N	4.0	21	2.64	1.58	94	0.79	94.6	215	368	5.73	244
First hospitalization												
3 (March 24th)	12.8	2.1	34	2.41	1.39	85	0.72	N	N	N	N	N
4 (March 25th)	6.7	3.4	27	2.18	1.10	84	0.76	94.0	218	275	2.80	51
7 (March 28th)	<1.5	12.5	43	5.30	3.95	103	0.83	95.4	283	193	1.89	37
10 (March 31st)	2.0	2.2	38	4.13	2.49	161	0.97	95.6	275	153	2.32	48
13 (April 3rd)	<1.5	0.8	60	3.62	2.34	217	1.00	96.0	261	110	1.61	35
15 (April 5th)	<1.5	0.3	N	4.08	2.47	225	1.16	96.9	218	82	N	N
31 (April 21st)	N	0.4	23	4.26	2.59	174	1.13	N	170	85	N	N
38 (April 28th):	N	0.9	38	6.06	4.69	176	0.86	97.8	199	128	N	N
First hospital discharge												
51 (May 11th):	N	N	N	N	N	N	N	N	N	N	N	N
Second hospitalization												
53 (May 12th)	N	0.5	21	4.69	3.08	173	1.17	97.6	182	105	N	N
81 (June 10th):	N	0.6	22	4.11	2.11	198	1.52	N	167	99	N	N
Second hospital discharge												
95 (June 24th):	N	0.7	36	4.72	2.98	213	1.4	96.9	196	133	N	N
Out‐patient follow‐up												
110 (July 9th):	2.0	0.3	31	4.95	3.35	155	1.15	97.7	223	262	3.69	44
Third hospitalization												
121 (July 20th)	N	0.7	27	4.84	3.06	226	1.26	97.4	203	13	1.45	25
128 (July 27th)	<1.5	0.5	20	4.91	3.07	224	1.36	97.0	169	118	N	N
149 (August 17th)	N	N	N	5.73	3.50	220	1.58	N	N	N	N	N
164 (September 1st):	<1.5	0.8	N	4.11	2.33	202	1.27	N	N	N	N	N
Third hospital discharge												
178 (September 14th)	N	0.7	33	4.91	3.36	193	1.03	96.6	205	375	N	N
194 (September 30th):												
Last follow‐up	N	0.9	23	4.13	2.73	191	0.97	96.5	218	366	N	N

Abbreviations: CK, creatine kinase; CK‐MB, creatine kinase isoenzyme; CRP, C‐reactive protein; ESR, erythrocyte sedimentation rate; IL‐6, interleukin‐6; LDH, lactate dehydrogenase; LYM, lymphocyte; MYO, myohemoglobin; N, absence of test; NEU, neutrophil; PLT, platelet; SaO_2_, arterial oxygen saturation; WBC, white blood cell.

* Reference of IL‐6 is 0–7 pg/mL; reference of CRP is <10 mg/L; reference of ESR is 0–20 mm/h; reference of WBC is 3.5–9.5×10^9^/L; reference of NEU is 1.8–6.3×10^9^/L; reference of PLT is 125–350×10^9^/L; reference of LYM is 1.1–3.2×10^9^/L; reference of SaO_2_ is ≥95%; reference of LDH is 115–220 U/L; reference of CK is 24–190 U/L; reference of CK‐MB is <3.61 ng/ml; reference of MYO is 28–72 ng/ml.

**TABLE 2:** Dynamics of T‐lymphocyte subgroups, total lymphocyte, and serum antibodies

Day of illness	CD3^+^ T‐LYM, cells/μL	CD4^+^ T‐LYM, cells/μL	CD8^+^ T‐LYM, cells/μL	Total LYM, cells/μL	CD4^+^/CD8^+^	IgM‐QD	IgG‐QD	IgG‐SQD
1 (March 22nd, 2020):	N	N	N	N	N	N	N	N
Disease onset								
2(March 23th):	639	302	303	753	1.0	N	N	N
First hospitalization								
7 (March 27th)	668	294	354	836	0.83	N	N	N
11 (April 1st)	807	437	347	997	1.26	N	N	N
13 (April 3rd)	848	476	362	1034	1.31	—	+	N
20 (April 10th)	1239	617	558	1542	1.11	—	+	N
24 (April 14th)	N	N	N	N	N	—	+	N
38 (April 28th):	N	N	N	N	N	N	N	N
First hospital discharge								
51 (May 11th):	N	N	N	N	N	—	+	N
Second hospitalization								
53 (May 13th)	1441	655	753	1752	0.87	N	N	N
62 (May 22th)	N	N	N	N	N	—	+	N
81 (June 10th):	1255	647	582	1506	1.11	—	+	1:10
Second hospital discharge								
95 (June 24th):	1274	599	645	1478	0.93	N	N	N
Out‐patient follow‐up								
110 (July 9th):	865	410	441	1193	0.93	N	N	1:1
Third hospitalization								
121 (July 20th)	1080	509	542	1303	0.94	—	+	1:1
139 (August 7th)	1395	680	679	1729	1.0	—	—	N
149 (August 17th)	N	N	N	N	N	—	—	N
164 (September 1st):	1055	475	554	1319	0.86	—	—	N
Third hospital discharge								
178 (September 14th)	908	399	485	1154	0.82	N	N	N
194 (September 30th):								
Last follow‐up	912	459	453	1112	1.01	N	N	N

Abbreviations: LYM, lymphocyte; N, absence of test; QD, qualitative detection; SQD, semi‐quantitative detection; +, positive for IgM or IgG against SARS‐CoV‐2; —, negative for IgM or IgG against SARS‐CoV‐2.

* Reference of CD3^+^ LYM is 955‐2860/μL; reference of CD4^+^ LYM is 550‐1440/μL; reference of CD8^+^ LYM is 320‐1250/μL; reference of CD8^+^ LYM is 1530‐3700/μL; reference of CD4^+^/CD8^+^ is 0.64–2.85.

**TABLE 3:** Dynamics of RT‐PCR test of SARS‐Cov‐2 RNA

Day of illness	Throat swab	Anal swab
1 (March 22nd, 2020): Disease onset 2 (March 23rd): First hospitalization 9 (March 30th) 14 (April 4th) 18 (April 8th) 21 (April 11th) 25 (April 15th) 34 (April 24th) 37 (April 27th) 38 (April 28th): First hospital discharge 51 (May 11th): Second hospitalization 58 (May 18th) 64 (May 24th) 69 (May 29th) 72 (June 1st) 76 (June 5th) 80 (June 9th) 81 (June 10th): Second hospital discharge 94 (June 23rd) 110 (July 9th): Third hospitalization 111 (July 10th) 118 (July 17th) 125 (July 24th) 129 (July 28th) 132 (July 31st) 146 (August 14th) 151 (August 19th) 153 (August 21th) 157 (August 25th) 163 (August 31st) 164 (September 1st): Third hospital discharge 177 (September 14th) 191 (September 28th): Last follow‐up	+ + + + + + + — — N + + + + + — — N — + + + — — + + — — — — N — —	N N N N N N N N N N N N N N N — — N N — — — — — — N — — — — N N N

Abbreviations: N, absence of test; +, positive for SARS‐CoV‐2 RNA; —, negative for SARS‐CoV‐2 RNA.

In this case, the patient was diagnosed with confirmed moderate COVID‐19 following diagnostic criteria,^1^ with a suspected early lung tumor and hypoxemia. She received antivirals with abidor and recombinant human interferon α‐2b spray, as well as other therapeutic drugs, including traditional Chinese medicine, ulinastatin, thymalfasin, human granulocyte colony stimulating factor, and amlodipine, among others, with administered oxygen inhalation via nasal cannula at 2L/minute. After two days of treatment, the patient developed cough and expectoration, although her fatigue disappeared and appetite improved. On illness Day 10 (nine days after initial positive virus test), the patient's condition became better with the disappearance of symptoms and the improvement of blood indexes (Tables 1 and 2) . A repeated chest CT revealed progression of partial lesions with absorption in both lungs (Figure 2B). On illness Day 13, serum antibodies against SARS‐CoV‐2 were detected based on gold immunochromatography (GICA) performed using a COVID‐19 antibody detection kit [Inot (Tangshan) Biotechnology Co., Ltd., China)], showing positive for IgG and negative for IgM. On illness Day 32, chest CT showed significant absorption of infected lesions in both lungs (Figure 2C). The following throat swab samples were continuously negative for SARS‐CoV‐2 RNA on illness Days 34 and 37, and the patient was finally discharged on illness Day 38. Following post‐discharge isolation management,^1^ the patient was transferred to the fixed isolation point for continuous solitary isolation and observation for the next 2 weeks.

On illness Day 51 (the 14th day of the isolation observation after discharge), the patient's throat swab was found to be RP for SARS‐CoV‐2 RNA, although she had no symptoms of discomfort. She was then transferred to our hospital once more for solitary isolation on that same day. The patient's anti‐SARS‐CoV‐2 IgG retest was positive, but her other laboratory results were normal. Chest CT this time showed continuous absorption of residual infection lesions in both lungs, and the suspected cancerous nodule in the LLL‐S^6^ was found to be slightly larger than before (1.2 × 0.9 cm). The patient was then given antiviral drug with recombinant human interferon α‐2b spray, immunopotentiator with thymalfasin, and hypotensive drugs with amlodipine and benazepril. Although her throat swab test was still positive for SARS‐CoV‐2 RNA on illness Day 72, she was continuously negative for SARS‐CoV‐2 RNA on illness Days 76 and 80. The detection of anti‐SARS‐CoV‐2 IgG was reduced to a weak positive, and the respective antibody titer was found to be 1:10 on illness Day 81. The patient kept well, and she was once again discharged for continuous solitary isolation and observation at the fixed isolation point for two weeks on illness Day 81.

The throat swab sample was rechecked with a negative SARS‐CoV‐2 RNA result 14 days after the second discharge, allowing the patient to finally go home for continuous solidary isolation and observation. Surprisingly, on illness Day 110 (the 16th day of home quarantine), the patient's throat swab was RP for SARS‐CoV‐2 RNA again, prompting transferal to our hospital for the third time despite being asymptomatic. Her chest CT on her third admission revealed a small number of residual infection lesions similar to previous findings; however, her initial solid nodule in the LLL‐S^6^ increased in size once more (1.4 × 1.0 cm). The re‐detection of SARS‐CoV‐2‐ IgG was found to be weakly positive, with an antibody titer reduced to 1:1 on illness Day 110 (Table 2). Elevated CK, CK‐MB, and LDH levels with reduced T‐lymphocyte subgroups were noted once again (Tables 1 and 2  and Figure 1), although tumor marker levels, serum 1,3‐β‐D‐glucan, galactomannan, and cryptococcus capsular antigen tests were all unremarkable. On her third admission, she was treated with thymalfasin for immuno‐enhancement, and hypotensive drugs with amlodipine and benazepril. Following treatment, LDH, CK, CK‐MB, and ESR levels all returned to normal. Additionally, T‐lymphocyte subgroups initially increased to normal, whereas the blood indexes declined again after a while. On illness Day 121, SARS‐CoV‐2‐ IgG titer remained at 1:1; however, anti‐SARS‐CoV‐2 IgG and IgM antibodies were both negative on illness Day 139 (Table 2). An additional GICA test was performed using a kit from a different manufacturer (Guangzhou Wondfo Biotech Co., Ltd.), showing negative IgG and IgM results. Although negative SARS‐CoV‐2 RT‐PCR results of throat swabs were found on illness Days 125 and 129, the collected samples showed RP once again on illness Days 132 and 146. Following this, the throat swabs were continuously negative for SARS‐CoV‐2 RNA on illness days 151, 153, 157, and 163. Furthermore, the re‐detections of anti‐SARS‐CoV‐2 IgG and IgM remained negative on illness Days 149 and 164. Meanwhile, repeated chest CT revealed few residual infection lesions similar to previous findings, but the initial solid nodule in the LLL‐S^6^ continuously enlarged (1.5 × 1.3 cm). On illness Day 164, the patient was discharged for continuous solitary isolation and observation for the third time at the fixed isolation point for 2 weeks, with subsequent home quarantine for the next 2 weeks. The latest outpatient follow‐up was done on September 30, 2020 (the 190th day of illness onset) when the patient remained well without signs of recurrence, except for slightly reduced T‐lymphocyte subgroups (Figure 1 and Table 2).

## 3 DISCUSSION

As seen in this case, the RP phenomenon for SARS‐CoV‐2 RNA lasted for about five months after disease onset. Although quantitative viral nucleic acid test, live virus isolation, and viral genome sequencing could not be performed due to the hospital's limitations, the case was considered to be prolonged viral shedding instead of a COVID‐19 re‐infection, which had also been reported in more than ten countries^15^ for several reasons as follows: (1) the patient was quarantined alone after the first discharge, including the isolation and observation period outside our hospital and the two re‐hospitalizations, while avoiding contact with any source of infection; (2) except for the slight increase of LDH, CK, CK‐MB, and ESR within a short time during the third hospitalization, important inflammatory markers, such as CRP and IL‐6, remained normal following the first hospital discharge; and (3) there were no new clinical symptoms or viral infection exacerbations found on dynamic chest CT after the first hospital discharge.

Prolonged viral shedding in this case was considered to be related to aging and accompanying underlying diseases which result in immune dysfunction,^2^, ^10^, ^12^, ^16^, ^17^ in addition to the insufficient antiviral effect of IgG antibodies. First, the solid nodule with lobulated sign and spiculation in the LLL‐S^6^ gradually increased in size within these 5 months while viral pneumonia had been obviously absorbed, indicating a high probability of tumor. Therefore, our elderly COVID‐19 patient had three underlying diseases, including hypertension, previous total hysterectomy for hysteromyoma, and a suspected lung tumor. Moreover, the total lymphocyte and T‐lymphocyte subset counts were found to be repeatedly decreased in dynamic monitoring. All aforementioned factors suggested cellular immune deficiency in this patient, which was an important consideration explaining the difficulty in completely eliminating the virus.^12^, ^18^ Second, anti‐SARS‐CoV‐2 IgG in this patient was initially tested with a positive result by qualitative detection on illness Day 13, whereas the antibody concentration was only 1:10 based on semi‐quantitative detection on illness Day 81 during the second hospitalization. This indicated IgG antibody titer in vivo was already extremely low at that time. Moreover, the IgG antibody titer continued to decline progressively to 1:1 on illness Day 110 during the third hospitalization, becoming undetectable as measured by two different tests on illness Day 139 even when the throat swab was still positive at that time. Although the peak of the IgG antibody titer in our patient was unknown at disease onset due to absence of quantitative detection, it was certain that the IgG concentration had been at a very low level about 2.5 months after onset, which was similar to previous reports.^19^, ^20^ Moreover, it was suggested the ability of the anti‐SARS‐CoV‐2 IgG antibodies decreased rapidly at the early course of the disease, nearly disappearing at the middle and later course of the disease in this case. Therefore, cellular immune deficiency and insufficient humoral immune response in the older patient resulted in a prolonged virus removal, as seen in this case. Additionally, the patient's immune status obviously fluctuated with frequent lymphocyte decreases throughout the whole disease course. Reduced immunity has been found to cause repeated increases in the number of viruses in the body, appearing as the phenomenon of intermittent virus replication.^21^ This may possibly explain the alternating positive and negative SARS‐CoV‐2 detection results in this patient.

This case suggests prolonged virus shedding is prone to occur in an immunocompromised host^5^, ^6^, ^7^, ^11^ since changes in the host immune status can lead to repeated positive SARS‐CoV‐2 detections. A recent case report has confirmed that SARS‐CoV‐2 can persistently survive with repeat replication for more than five months after initial infection,^6^ suggesting that some cases with prolonged virus shedding could be associated with prolonged infectivity. Therefore, for such patients, it is necessary to increase the frequency of SARS‐CoV‐2 nucleic acid testing and enhance post‐discharge isolation management and health monitoring.^1^ Notably, the IgG antibody titer in this case decayed rapidly at the early course of disease onset, and the antibody completely disappeared in less than five months before virus removal, indicating that there may be certain limitations on the protective effect of anti‐SARS‐CoV‐2 antibodies, especially in immunocompromised hosts.

## CONFLICT OF INTEREST

The authors declare that there is no conflict of interest.

## AUTHOR CONTRIBUTIONS

JH conceived the study, drafted the study, and reviewed all drafts of the study. CL managed the data generation, data analysis, and drafted the study. XW helped to carry out clinical data collection. MH helped to carry out laboratorial data collection. JH and CL contributed equally as senior authors. All authors read and approved the final study.

## Data Availability

The data used to support the findings of this study are available from the corresponding author upon request.
